# The role of the insula predicting memory performance in Alzheimer's disease amyloid positive and negative: a clinical cohort study

**DOI:** 10.1002/alz.087942

**Published:** 2025-01-09

**Authors:** Michelle M Coleman, Marc W Haut, Camila Vieira de Ligo Teixeira, Cierra M Keith, Rashi Mehta, Holly Phelps, Melanie Ward, Mark Miller, R. Osvaldo Navia, Gary D Marano, Xiaofei Wang, Stephanie Pockl, Nafiisah Rajabalee, William T McCuddy, Pierre‐Francois D’Haese, Kirk Wilhelmson

**Affiliations:** ^1^ Rockefeller Neuroscience Institute, West Virginia University, Morgantown, WV USA; ^2^ West Virginia University, Morgantown, WV USA; ^3^ St. Joseph Hospital and Medical Center, Phoenix, AZ USA

## Abstract

**Background:**

Known areas of Alzheimer’s pathology, including the hippocampus, entorhinal cortex and medial temporal cortex, have been well‐demonstrated as demonstrating atrophy in Alzheimer’s disease (AD). Using surface‐based morphometry measures, recent studies have suggested that the insula may play a role in memory function. Differences in patients based on amyloid biomarkers are increasingly being studied, particularly comparing individuals who were clinically diagnosed as AD but have negative amyloid biomarkers with those who are positive for amyloid. Atrophy may be similar in the entorhinal cortex between individuals who are amyloid positive versus amyloid negative, despite differences in memory. Here, we aimed to investigate the role of the insula in memory in a cohort of patients (aMCI and dementia) who were positive for amyloid with those who were negative for amyloid.

**Method:**

We retrospectively analyzed clinical data from 161 aMCI and dementia patients (A+ = 106, A‐ = 55) and 41 healthy participants. Participants completed MRI and cognitive measures. Analysis of cortical thickness and gyrification index of the AD signature areas and the insula were completed using CAT12 and the DKT40 atlas. Memory performance was assessed using the California Verbal Learning Test short form, with long‐delay‐free recall as the variable of interest. Pearson’s correlations and stepwise linear regression were run using signature areas' significant correlations and interactions (adjusted p<0.001).

**Result:**

Significant correlations with memory performance included insula gyrification and entorhinal and inferior parietal thickness (r=.25, r=.23, and r=.22, respectively). The interaction of insula gyrification with entorhinal thickness demonstrated significant results in predicting memory performance, accounting for 11% variance, even controlling for age. The interaction of reduced entorhinal cortex thickness and increased insula gyrification best explains delayed recall.

**Conclusion:**

In our well‐characterized clinical cohort of aMCI and dementia with positive and negative amyloid biomarkers, insula gyrification interaction with entorhinal thickness best predicted memory performance measured by CVLT long delay. This finding suggests that, regardless of amyloid status, reduced entorhinal cortex thickness and increased insula atrophy (measured by gyrification, not thickness), impact delayed memory. Studies are necessary to understand the insula's function in memory as well as gyrification and thickness variations across brain regions and their role in cognition.

